# Data Note: Linking Shopping Transaction Data into the Avon Longitudinal Study of Parents and Children

**DOI:** 10.12688/wellcomeopenres.27761.1

**Published:** 2026-10-08

**Authors:** Romana Burgess, Mark Mummé, Andy Boyd, Anya Skatova

**Affiliations:** 1 Digital Footprints Lab, Population Health Sciences, University of Bristol Medical School, Bristol, England, UK; 2 The MRC Integrative Epidemiology Unit, Population Health Sciences, University of Bristol Medical School, Bristol, England, UK; 3 ALSPAC, University of Bristol Medical School, Bristol, England, UK; 4 UK Longitudinal Linkage Collaboration, University of Bristol, Bristol, UK; 5 Health Data Research UK, London, England, UK

**Keywords:** Shopping Data; Data Linkage; Loyalty Cards; Longitudinal Population Study; ALSPAC

## Abstract

**Introduction:**

This data note describes the collection and linkage of participant’s Sainsburys shopping data into the Avon Longitudinal Study of Parents and Children (ALSPAC).

**Methods:**

Data were collected via informed participant consent and Subject Access Requests under UK GDPR, securely transferred and integrated into the ALSPAC database using unique study identifiers. Participant consultation and a technical pilot ensured transparent, acceptable, and secure linkage.

**Results:**

The dataset contains anonymised transactions for 244 participants, covering 658,375 item purchases between July 2014 and February 2024. Transactions include an item description, price, quantity, time, date, store location, and other variables. Items fall into 82 categories (e.g., produce) and 1002 subcategories (e.g., bananas). In terms of representativeness, the cohort is skewed towards females, consists of participants of similar age, and is predominantly made up of individuals with higher educational attainment and of White ethnicity.

**Conclusion:**

The linkage of shopping records complements ALSPACs extensive existing dataset, which comprises over 30 years of sociodemographic and health-related data. The linkage stands as a novel opportunity for future research into dietary habits, nutritional intake, and subsequent health outcomes. Access to data may be granted to approved researchers upon reasonable request, following a proposal submission through the ALSPAC website ( http://www.bristol.ac.uk/alspac).

## Key features

• This dataset represents a pioneering linkage of supermarket transaction records into a longitudinal cohort study, creating a novel opportunity to explore relationships between purchasing and health and social outcomes.
• The dataset covers 244 participants, including more than 658,000 item purchases over a ten year period.
• Each purchase is associated with an item description, the time and date of purchase, the quantity and price of the item bought, the product category, and store location, among other variables.
• The shopping records complement the extensive existing data collected by the Avon Longitudinal Study of Parents and Children (ALSPAC), allowing future research to explore associations with health, lifestyle, environmental and social factors.
• The data can be accessed through the study’s Trusted Research Environment to approved researchers upon reasonable request; interested parties may submit a proposal through the ALSPAC website ( http://www.bristol.ac.uk/alspac).

## Background

Supermarket loyalty card data offer an objective, large-scale approach for studying purchasing behaviour over time in a real-world setting. By capturing a range of products—such as food and drink, alcohol and tobacco, lottery tickets, healthcare and medications—there is scope for these data to inform public benefit research across a variety of contexts. ^1^ So far, most studies have investigated questions relating to dietary patterns, such as protein consumption ^2^ or dietary diversity within a population ^3^, or nutritional intake,including deficiencies linked to dietary transitions ^4^ or deprivation. ^5^ Many have also investigated sociodemographic inequalities in access to healthy foods, ^6^ ^,^ ^7^ while others have tracked self-medication use, ^8^ in particular, as early indicators of cancer. ^9^ Others have evaluated the dietary impacts of supermarket-based interventions or policy changes (e.g., the soft drink sugar tax) ^10^ or alcohol legislation reform). ^11^ This wide variety of topics exemplifies the huge potential for health research presented by these data.

However, standalone transactions provide limited context to purchasing behaviour, ^12^ making linkages with other data sources (e.g., cross-sectional surveys and clinical measurements) essential for deeper investigation. As a result, attention has turned to integrating these records into longitudinal population studies. ^13^ This approach allows researchers to triangulate insights from shopping data with the rich demographic and self-report data offered by longitudinal studies, offering a more comprehensive overview of participant behaviour. These linkages not only enrich longitudinal population studies, but also allow for the assessment of biases and limitations within the transaction data.

This data note describes a linked dataset, integrating Sainsburys transaction data into a UK longitudinal population study: the Avon Longitudinal Study of Parents and Children (ALSPAC), also as known as “Children of the 90s”. Sainsbury’s is one of the largest supermarket chains in the United Kingdom, operating a nationwide network of grocery stores. In 2002, Sainsbury’s introduced the Nectar loyalty card, consolidating earlier loyalty schemes into a single points-based system. ^14^ By the mid-2010’s, the Nectar Card was one of the UK’s largest loyalty programs, with millions of active users; Sainsbury’s share of the UK grocery retail market during this period fluctuated between 15% and 17%. ^15^

We describe the collection of Sainsbury’s data and subsequent linkage into the ALSPAC database, and give an overview of the integrated dataset.

## Methods

The preprint for this work has been shared previously. ^16^

### The avon longitudinal study of parents and children

The Avon Longitudinal Cohort Study of Parents and Children (ALSPAC) is a longitudinal population study based in Bristol, UK. ^17^ ^–^ ^19^ Initial recruitment of women resident in (the historical county of ) Avon took place between September 1990 to December 1992, recruiting 14,541 pregnancies with expected dates between April 1 ^st^ 1991 and 31 ^st^ December 1992. This resulted in 14,062 live births, of which 13,988 infants were alive at one year after birth. ^17^ The original pregnant women and their partners are referred to as Generation 0 (G0), ^18^ and their children—the ALSPAC index participants—are referred to as Generation 1 (G1). Further recruitment has resulted in 913 additional G1 participants joining since age 7, including 195 from age 18. ^20^ The total sample size for analyses using any data collected after the age of seven is therefore 15,447 pregnancies, resulting in 15,658 foetuses. Of these, 14,901 children were alive at 1 year of age.

Of the original 14,541 initial pregnancies, 338 were from a woman who had already enrolled with a previous pregnancy, meaning 14,203 unique mothers were initially enrolled in the study. As a result of the additional phases of recruitment, a further 630 women who did not enrol originally have provided data since their child was 7 years of age. This provides a total of 14,833 unique women (G0 mothers) enrolled in ALSPAC as of September 2021. G0 partners were invited to complete questionnaires by the mothers at the start of the study and they were not formally enrolled at that time. 12,113 G0 partners have been in contact with the study by providing data and/or formally enrolling when this started in 2010. 3,807 G0 partners are currently enrolled.

The participants in the linked supermarket dataset are all from the G1 cohort, meaning that they are all approximately the same age.

Over 30 years, ALSPAC has routinely collected participants’ phenotypic, environmental, genetic, and biological data through clinics, questionnaires, and linked records (e.g., primary care, mental health services, education and crime records). This has led to an extremely rich dataset, making ALSPAC a valuable resource for health research. In addition, comprehensive demographic data help to mitigate biases by allowing researchers to control for socioeconomic factors. The study website contains details of all data available through a fully searchable data dictionary and variable search tool ( http://www.bristol.ac.uk/alspac/researchers/our-data/).

Study data are collected and managed using REDCap electronic data capture tools hosted at the University of Bristol ^21^; REDCap (Research Electronic Data Capture) is a secure, web-based software platform designed to support data capture for research studies.

### Participant consultation

Extensive engagement and involvement with the ALSPAC cohort allowed us to design a secure, ethically compliant, and trusted approach for collecting and linking participant’s supermarket data, built on participant input.

In 2021, semi-structured focus groups were held to ask 20 participants about their attitudes to donating and linking transactional data for research. ^22^ In these sessions, participants highlighted understanding (of the research purpose), trust (in researcher motivations), and control (of who uses their data and what for) as key themes. A subsequent summary report, ^23^ which included this study alongside related work, summarised participant views on the broader acceptability of collecting their “digital footprint” data. This report found that digital footprint data collection was perceived to be acceptable to the ALSPAC cohort, subject to clear consenting strategies, rigorous governance by trusted data custodians, and transparent data processing. In the case of loyalty card data, these transactions were found to be much less sensitive in context compared to other data types, such as GPS or search history. Finally, a recent interview study ^24^ asked twelve participants to download their data for discussion with researchers. Throughout the interviews, no concerns were raised regarding the sensitivity of shopping data, or surrounding any specific variables (e.g., store location, particular products).

We later launched a survey which asked participants (n = 3768) whether they owned a loyalty card from any of five listed supermarket brands, and whether they would likely consent to linking these data into ALSPAC in the future. ^24^ Overall, 63% of participants expressed that they owned a loyalty card for one of the five retailers; 92% of these indicated that they would share their transaction data. This included 1141 owners of Sainsburys Nectar Cards.

The collection and linkage of shopping data was approved by the ALSPAC Ethics and Law Committee on the 26 ^th^ July 2022 (Project ID: B3200). Informed consent for the use of all data collected was obtained from participants following the recommendations of the ALSPAC Ethics and Law Committee at the time. Participants can contact the study team at any time to retrospectively withdraw consent for their data to be used. Study participation is voluntary and during all data collection sweeps, information was provided on the intended use of data.

The protocol for this work can be found elsewhere. ^25^

### Technical pilot

To test secure transfer of shopping data from the supermarket to ALSPAC, we conducted a technical pilot with ten participants. Data were retrieved as “Subject Access Requests” under the UK GDPR. Sainsburys reviewed the participant documentation and approved its suitability. ALSPAC sent individual documents for each participant, including their name (as on the loyalty card), participant reported Nectar card number, and electronic signature. Files were encrypted, password protected and sent via the university’s secure data transfer system, “FLUFF” (Facility for the Upload of Large Files). The first document was sent on April 6 ^th^ 2023, to confirm receipt accuracy; the remaining nine were sent on April 13 ^th^. Sainsburys returned all files on 10 ^th^ May, through their own secure, password-protected system. Data arrived as ten separate Excel spreadsheets, split into transaction and summary level records—2,654 transactions from 2013 onwards, covering 33,452 items over 69 categories (or 659 sub-categories). This pilot confirmed secure and accurate data transfer, laying the foundation for the full linkage.

## Results

### Data collection and linkage

Between September and November 2023, 6170 participants were invited by email to share their loyalty card data with ALSPAC. Only participants for whom ALSPAC held an active email address were contacted (i.e., no paper-based or postal consent was pursued). A total of 511 participants positively responded to the request. As part of the invitation, participants were asked whether they held loyalty cards for any of five major UK supermarket schemes—Tesco Clubcard, Sainsbury’s Nectar, Boots Advantage, Morrisons More, and Co-op Membership—and were then invited to provide informed consent for ALSPAC to request their loyalty card data from these retailers. These five supermarkets were selected because, at the time that ethical approval was obtained, they represented some of the largest UK loyalty card schemes.

It is important to note that we do not know how many of the 6,170 contacted participants actually held loyalty cards, as this information was only collected from those who chose to respond.

Of the 511 respondents, 457 reported owning at least one loyalty card and provided details for one or more of these schemes. The number of consenting participants per retailer was as follows: Tesco (n = 363), Sainsbury’s (n = 263), Boots (n = 200), Morrisons (n = 55), and Co-op (n = 66). More information can be found elsewhere. ^24^ Sainsbury’s was selected for initial linkage, as the sample size was one of the largest and the retailer was receptive to data sharing. Data collections from the remaining supermarkets are still in progress and will be incorporated into future updates of this work.

Sainsbury’s provided the data under the UK GDPR via Subject Access Requests, having approved the participant facing materials and engaging with this project due to the perceived public benefits. ALSPAC sent each participants details—including name (as on the card), card number, and electronic signature—as encrypted, password-protected documents using the university’s secure transfer system (FLUFF). Sainsburys returned the data on 20 ^th^ February 2024, similarly encrypted. Raw data were received as individual Excel files, with one file per participant. A script was written (by author M.M) to compile these into a single dataset. Free-text item descriptions were converted into numeric variables for analysis. No data were removed during processing, though additional protections (e.g., small cell compression) may be later applied within the ALSPAC the Trusted Research Environment (TRE) where the data are stored.

In total, data were retrieved for 244 participants (see Figure 1). Linkage into the ALSPAC database was carried out by the ALSPAC data linkage team, matching records using study IDs.

**Figure 1.**
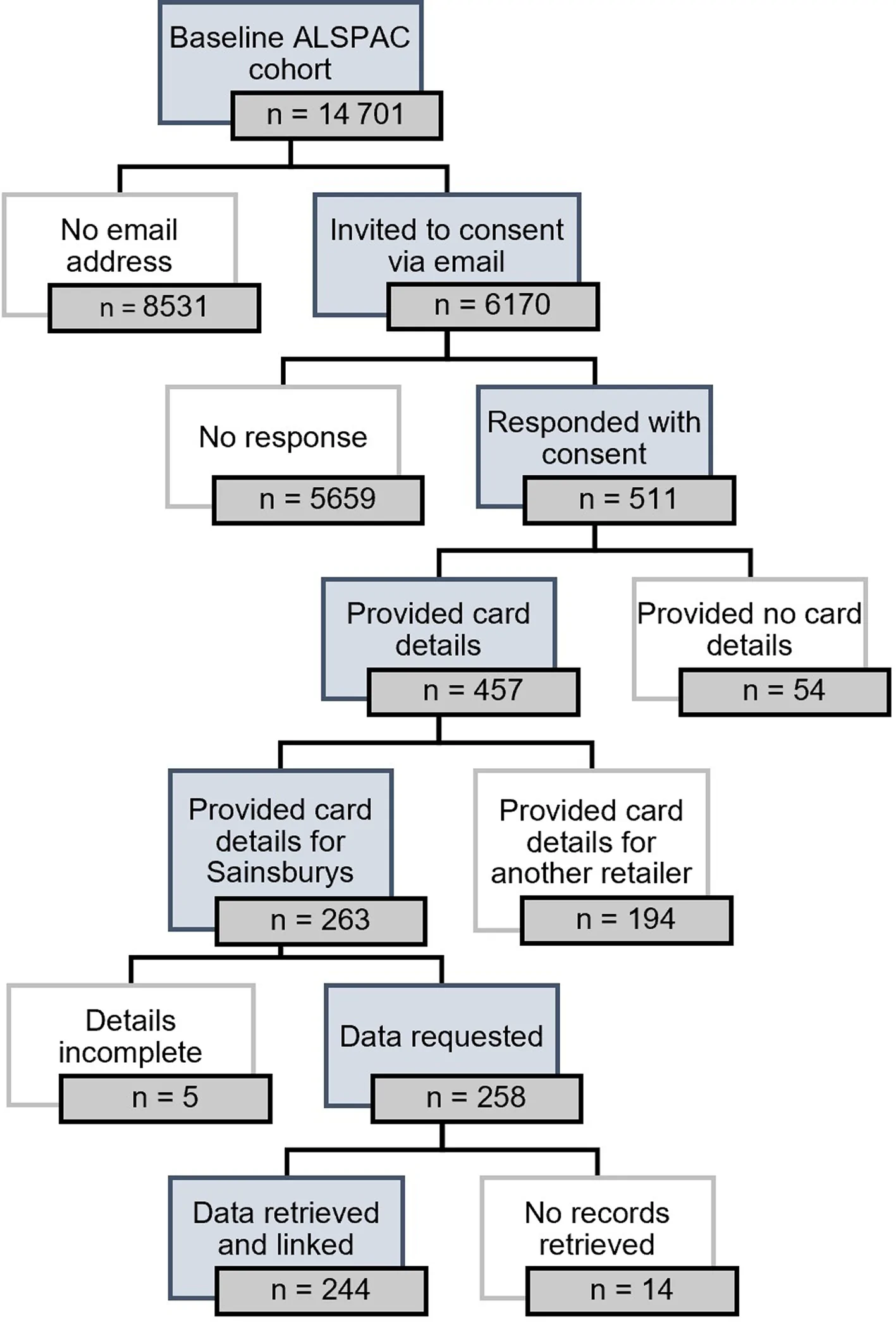
Flow chart to show number of participants at each stage of the Sainsbury’s data collection and linkage process.

### Representativeness of the linked sample

We present the demographics of the linked participants compared to the wider G1 cohort, demonstrating that the linked cohort is largely representative aside from small differences in financial comfort and educational attainment. For these comparisons, we use a range of measures that are temporally aligned with the supermarket data linkage, and proportions are calculated based solely on the participants who provided data for each measurement. In essence, this means that we compare to the recent typically-responding G1 participants.

The average age of the linked participants, all members of G1, was 30.9 years (SD = 0.6) in April 2023. The linked cohort is 63% female (153/244), compared to 45% in the wider G1 cohort (14,859/33,419). In addition, 97% of the linked participants are of white ethnicity compared to 95% of the wider G1 cohort (11,447 of 12,055), which broadly represents the ALSPAC catchment area at the point of enrolment. ^17^

Table 1 shows the highest educational attainment of the linked supermarket cohort and wider G1 cohort. In total, 188 participants provided educational data in the linked cohort, and 3576 participants provided these data in the wider G1 cohort. The number of participants with a degree and above is comparable across cohorts, however, the number of participants whose highest educational attainment is GCSE and equivalent is notably fewer in the linked cohort (and as such, there are more participants with A levels and equivalent).

**Table 1.** Highest educational attainment for the linked supermarket cohort and the wider G1 cohort.

Highest educational attainment	Linked supermarket cohort (n = 188)	Wider G1 cohort (n = 3576)
Degree and above	85 (45%)	1710 (48%)
A level and equivalent	81 (43%)	1230 (34%)
GCSE, O level, and equivalent	22 (12%)	636 (28%)

Table 2 shows the pre-pandemic employment status of the linked supermarket cohort and wider G1 cohort. In total, 220 participants provided employment data in the linked cohort, and 2449 participants provided these data in the wider G1 cohort. The numbers of participants are comparable across cohorts, with the vast majority being in full-time employment (around 85%), and many also working part-time (around 15%). Fewer participants are in full-time education or unemployed (less than 10% and 5% respectively).

**Table 2.** Pre-pandemic employment for the linked supermarket cohort and the wider G1 cohort.

Employment status	Linked supermarket cohort (n = 220)	Wider G1 cohort (n = 2449)
Full time or self-employment	190 (86%)	2024 (83%)
Part time/irregular employment	31 (14%)	367 (15%)
Full/part time education	18 (8%)	231 (9%)
Unemployed/unable to work	9 (4%)	96 (4%)
Other (e.g., apprenticeship, care)	38 (17%)	307 (13%)

Note that participants may appear in multiple categories.

Table 3 shows the pre-pandemic financial status of the linked supermarket cohort and wider G1 cohort. In total, 225 participants provided their financial status in the linked cohort, and 2571 participants provided these data in the wider G1 cohort. There are some differences in financial comfort between cohorts, with a lower percentage of the linked participants expressing that they were living comfortably compared to the wider G1 cohort, and more participants responding as doing “all right”, “just about getting by”, or “finding it difficult”.

**Table 3.** Pre-pandemic financial status for the linked supermarket cohort and the wider G1 cohort.

Financial status	Linked supermarket cohort (n = 225)	Wider G1 cohort (n = 2571)
Living comfortably	70 (31%)	1188 (46%)
Doing all right	108 (48%)	1035 (40%)
Just about getting by	33 (15%)	277 (11%)
Finding it quite or very difficult	14 (6%)	71 (3%)

### Description of the linked data

The dataset spans between July 2014 and February 2024, and includes 68,392 transactions—658,375 items in total—from 244 participants who consented to linking their Sainsbury’s data. The dataset comprises 24 variables, including: item description, price, quantity, date and time, product category and subcategory, store code, regional code (see Table 4 for full metadata).

**Table 4.** Variables in the shopping data.

Variable	Description	Example/format
ID	Participant ID number	14-digit number
Gender	Participant gender	f/m
Transaction Date	Date of purchase	yyyy-mm-dd
Transaction Time	Time of purchase	hh-mm-ss
Store Index	Store ID	Numeric value
Item Description	Item name	String, e.g., JS GARLIC & CORIANDER MINI NAANS X4 188G
Subcategory Index	Subcategory name	String, e.g., Indian
Category Index	Category name	String, e.g., Packaged Grocery
Item Weight/Volume	Weight or volume of item	Numeric, e.g., 188
Weight/Volume Index	Unit for item volume or weight	String, e.g., G/ML
Quantity Per Item	Quantity of items in transaction	Numeric, e.g., 12
Unit price	Cost of single unit within item (e.g., 1 beer in a 6 pack)	Numeric (£), e.g., £1.50
Amount Spend per Item	Total cost of item	Numeric (£), e.g., £1.50
Total Items Quantity	Total items in transaction	Numeric, e.g., 3
Total Transaction Value	Cost for entire transaction	Numeric (£), e.g., £20.45
Discount Rate	Discount (if applied)	Percentage, e.g., 0.20
Transaction Item	Nth item scanned during transaction	Numeric, e.g., 1
OA	ONS Output Area of store	Nine-digit letter/number combination corresponding to an area
LSOA	ONS Lower Layer Super Output Area of store	Nine-digit letter/number combination corresponding to an area
MSOA	ONS Middle Layer Super Output Area of store	Nine-digit letter/number combination corresponding to an area

Figure 2 illustrates the key features of shopping data and the links between them. The features—transactions, items, categories, and people—are distinct elements, each of which can be analysed across various dimensions, including time, spend, location, and quantity. Items are the most granular level of the data, forming a subset of both categories and transactions. People can encompass not only individual card holders, but also households (i.e., if card holders shop for other people). Note that transactions are sometimes referred to as “baskets” in the literature.

**Figure 2.**
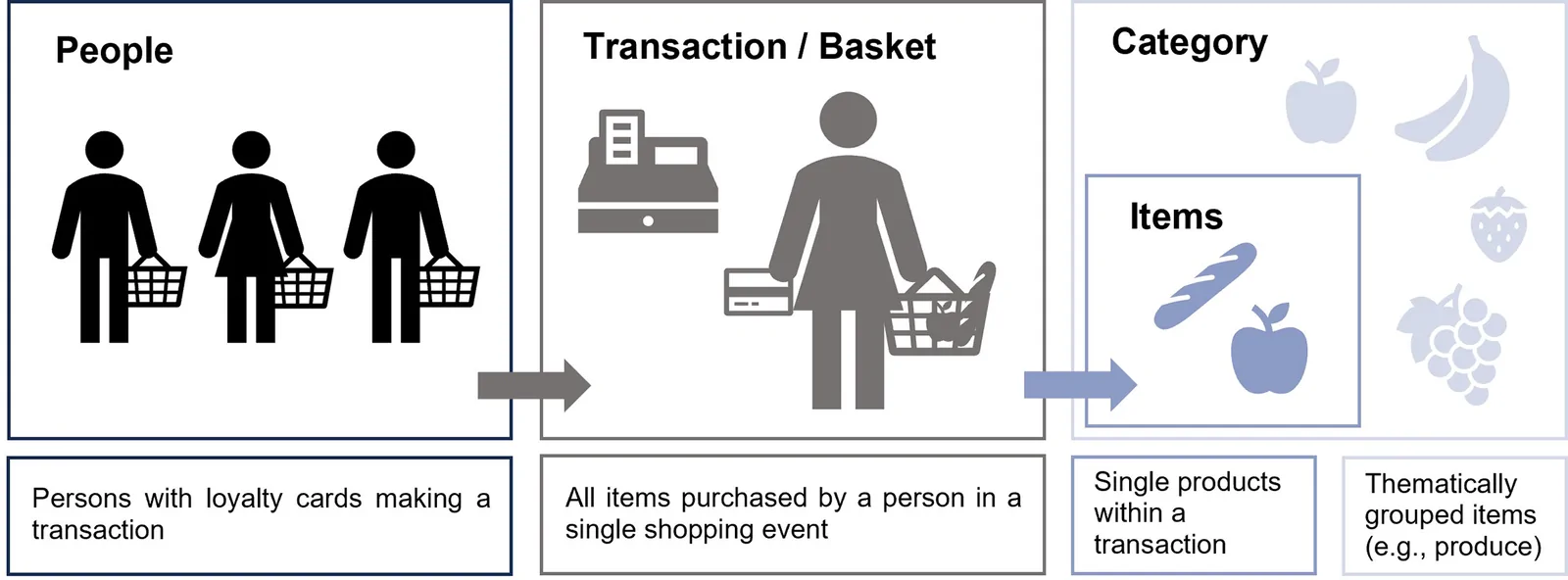
Key features of shopping data.

Data covers a ten year period (2014–2024), corresponding to the span of records available for linkage. Figure 3 shows the number of active cards by calendar year (i.e., the number of cards associated with transactions in a given year); the number of active cards increases each year. Note that 2024 is excluded as data were incomplete for that year. For instance, there were 90 active cards in 2014, and 234 in 2023. On average, participants used their cards for 2054 days (SD = 1256.13), or around 5.6 years. The minimum is 1 day, and the maximum is 3507 days (~9.6 years). As such, most card owners in the linked dataset used their card over multiple years.

**Figure 3.**
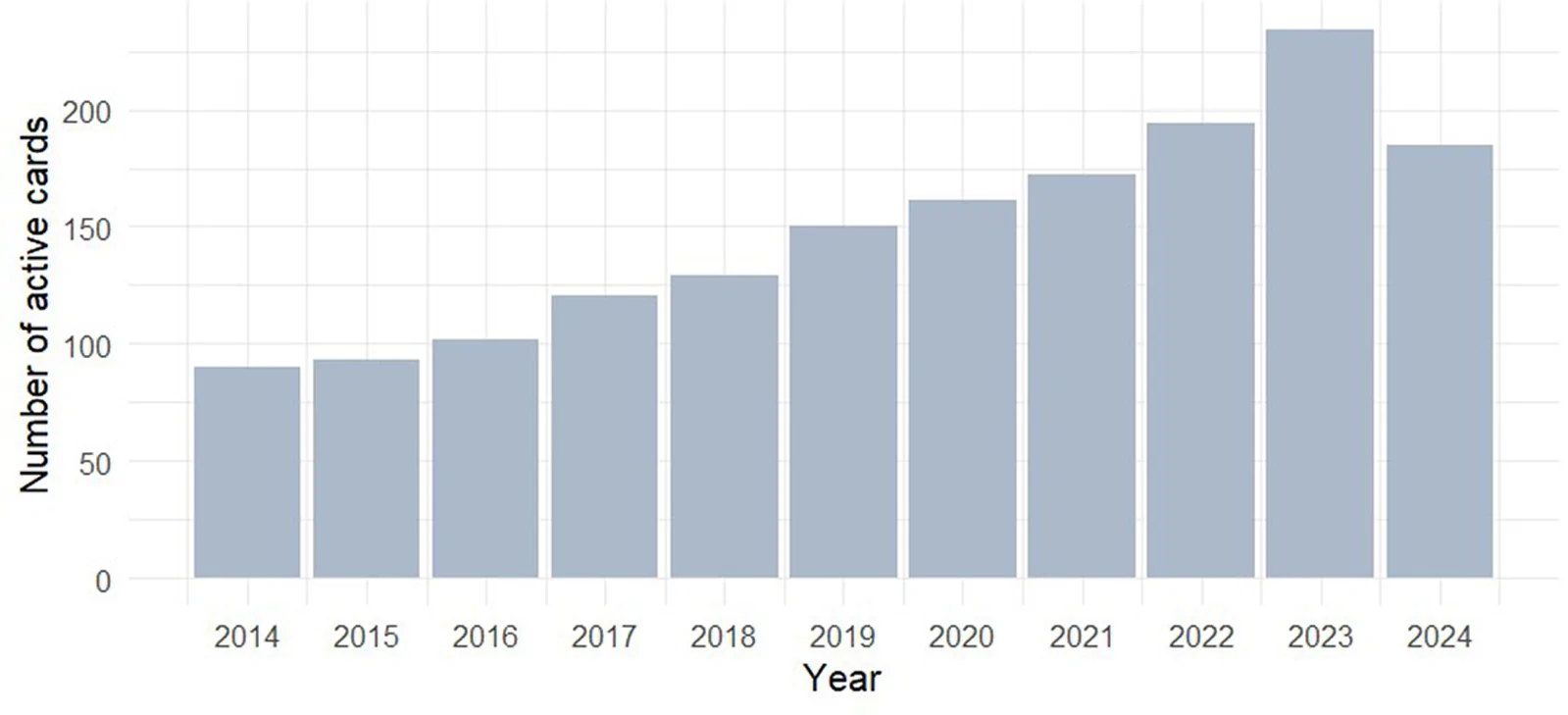
Number of active cards by calendar year.

### Transactions

Figure 4 shows a histogram of transactions by participant. The mean number of transactions by a single participant is 280.09 (SD = 317.5); the fewest number of transactions is 1 (i.e., one shopping event), and the maximum is 2075. This reflects a large variety of shopping activity, with most participants using their loyalty card during a few hundred shopping trips.

**Figure 4.**
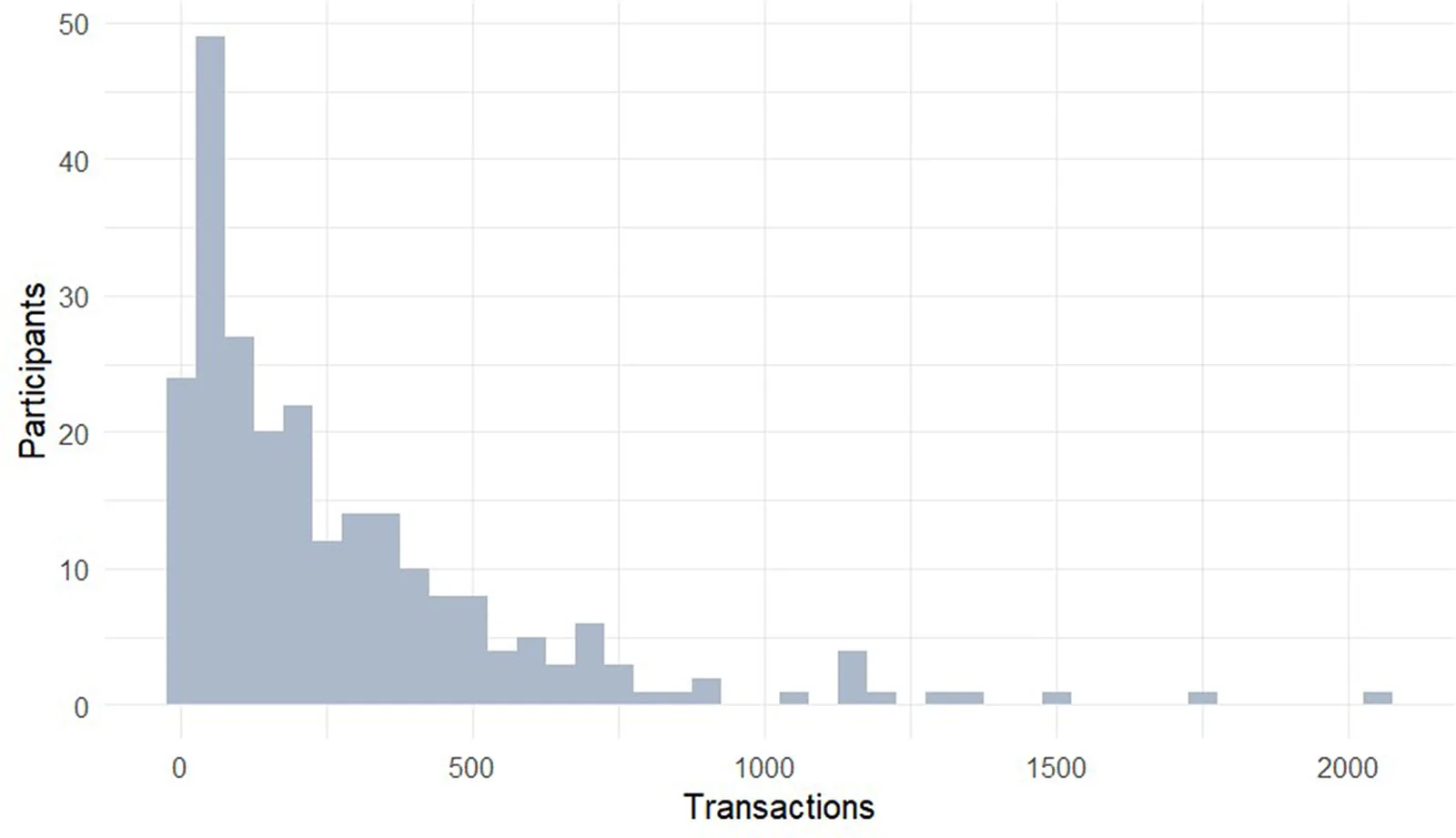
Frequency of transactions by participant.

Table 5 shows the total spend per transaction, grouped into categories; transactions of £0 or less represent refunds. Most transactions total between £1 and £5 (26%) or £21 and £50 (24%), while larger transactions—greater than £100—are less frequent (2%). As such, transactions range from small, convenience purchases to much larger food shops. The average transaction costs £22.41 (SD = 27.70), with the maximum transaction spend being £749.96.

**Table 5.** Total value of transactions, grouped categorically.

Transaction Value (£)	Frequency	Percentage (%)
<£0	722	1.13
£1–£5	17,775	25.99
£6–£10	12,282	17.96
£11–£20	12,474	18.24
£21–£50	16,384	23.96
£51–£100	7482	10.94
£101+	1223	1.79

### Items

Figure 5 shows the total number of items purchased by year, alongside total spend. Both variables increase steadily each year, with a rapid increase between 2022 to 2023. This likely reflects the increase in active card users over time (see Figure 3), but may also reflect price inflation and changes in household composition and/or needs (e.g., births of children). The dataset therefore captures not only individual snapshots of shopping activity, but also longitudinal economic and behavioural shifts.

**Figure 5.**
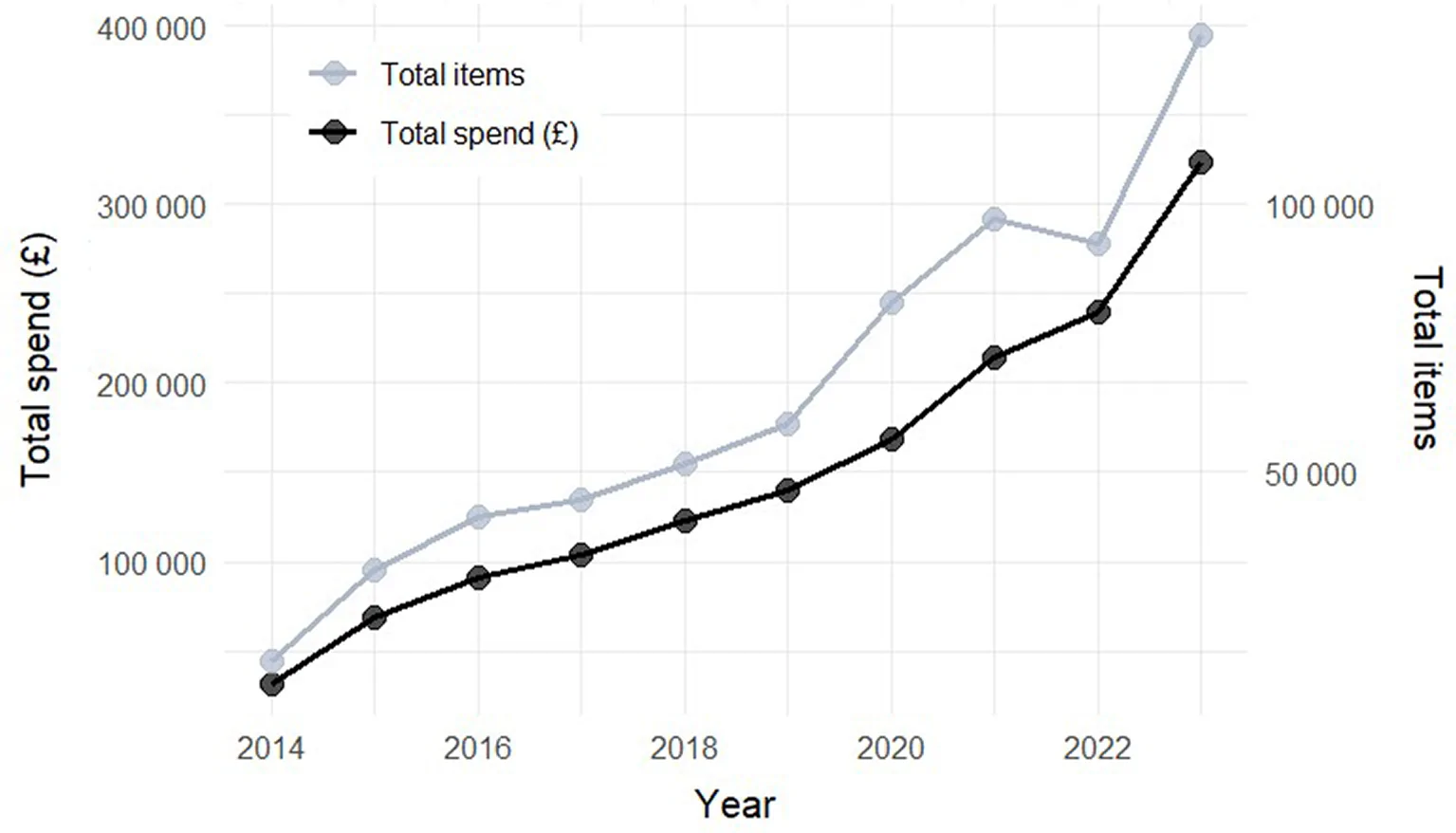
Total items purchased and spend by year.

Figure 6 shows a histogram of total items purchased by participant. The mean number of items purchased by a single participant is 2698.26 (SD = 3222.45); the fewest number of items purchased is 2, and the maximum is 17,871. This reflects a variety of card users—from highly active to occasional users.

**Figure 6.**
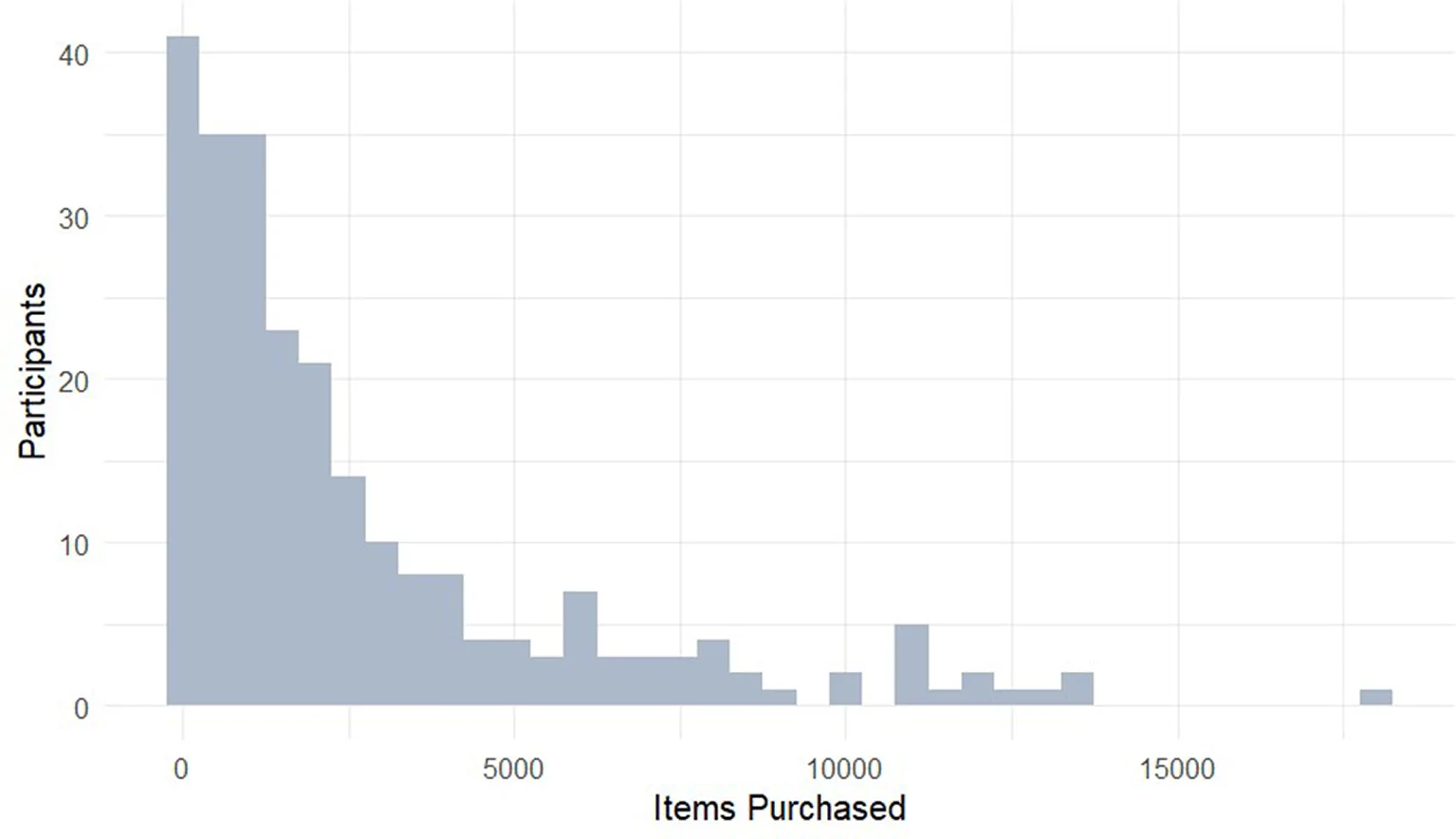
Frequency of items purchased by participant.

Table 6 shows the total numbers of items within a transaction, grouped categorically. Most transactions contain fewer than ten items, while transactions containing over 100 items are rare. Transactions containing a negative number of items (<0) represent refunds. The average transaction contains 9.63 items (SD = 13.88), with the largest refund being for 44 items, and the largest transaction containing 272 items.

**Table 6.** Number of items per transaction, grouped categorically.

Items per Transaction	Frequency	Percentage (%)
< 0	724	1.06
1–5	38,160	55.84
6–10	11,753	17.20
11–20	8822	12.91
21–50	7257	10.62
51–100	1523	2.23
101+	103	0.15

Figure 7 shows the total items purchased by month, week day, and hour. On a monthly basis, purchases are fairly consistent, although there are some increases towards the end of the year (towards Christmas, where consumption typically increases) and a notable decrease in February (possibly because the month is the shortest). Most purchases occur on a Monday, followed by Friday and Saturday, with the fewest purchases occurring around midweek. This likely reflects weekly household shops (Monday), and visits during free time (the weekend). Purchases by hour reveal that most purchases are made around midday (12 to 1 pm) and in the early evening (5 to 6 pm)—consistent with lunch breaks and post-work shopping trips—with much fewer purchases made late at night and in the early morning. Together, these patterns support research into routine behaviours and the influence of work schedules and family responsibilities on purchasing.

**Figure 7.**
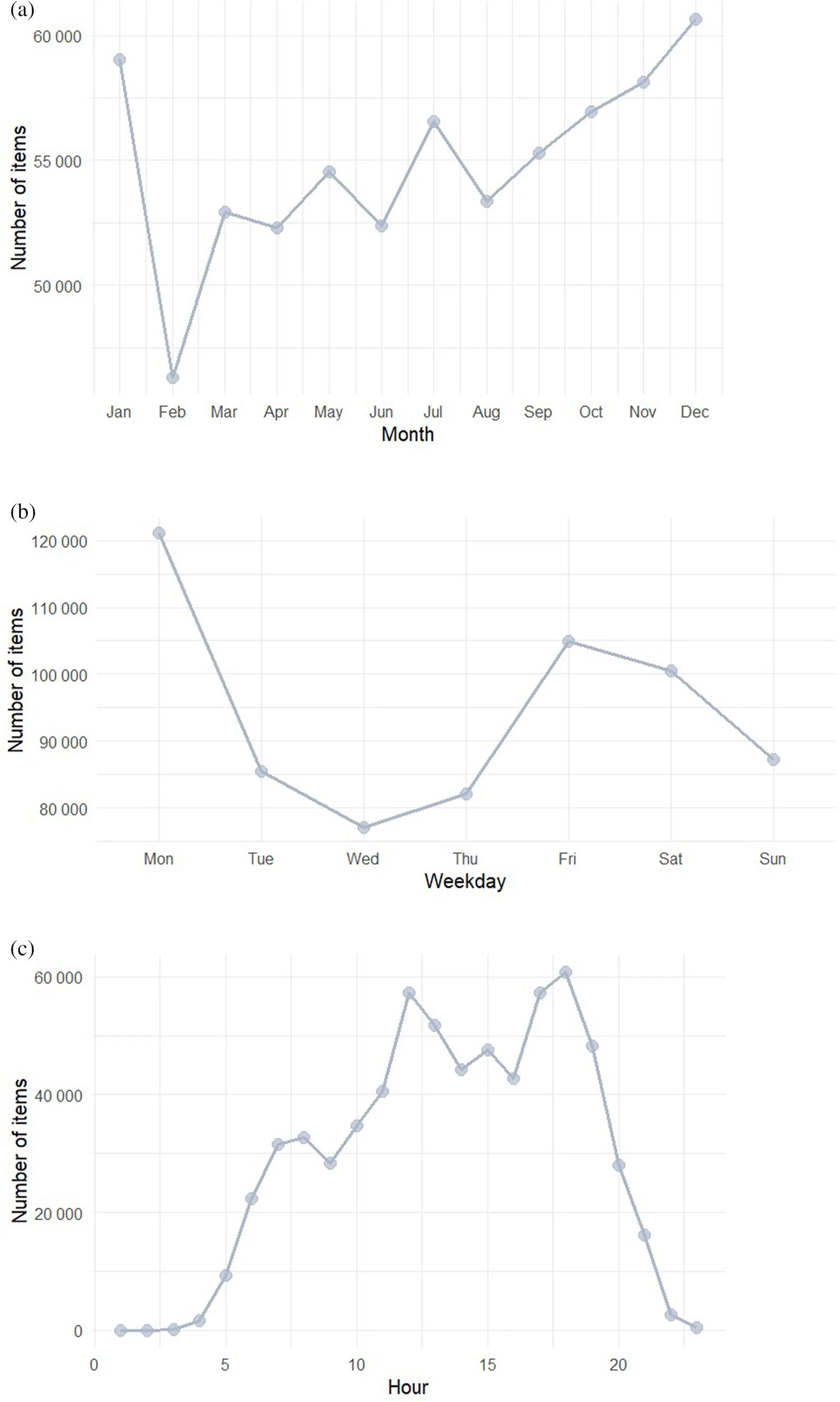
Total number of items purchased by a) month, b) week day, and c) hour.

### Spend

The total spend across the dataset is £1,531,686, reflecting the large scale of the data. On average, participants spent £6277.40 in total (SD = 7225.86); the lowest cumulative spend by a participant is £3.20, and the maximum is £49,050.28.

Figure 8 shows participant’s average spend by year, month, and day of the week. Increased spending over time likely reflects price inflation and evolving household compositions (i.e., children). Spending is highest in December, and lowest in February, with another increase during the Spring months. Finally, spending is highest on the weekend, followed by Monday and Friday, with the lowest spending occurring around midweek—this likely reflects routine restocking and household shopping trips.

**Figure 8.**
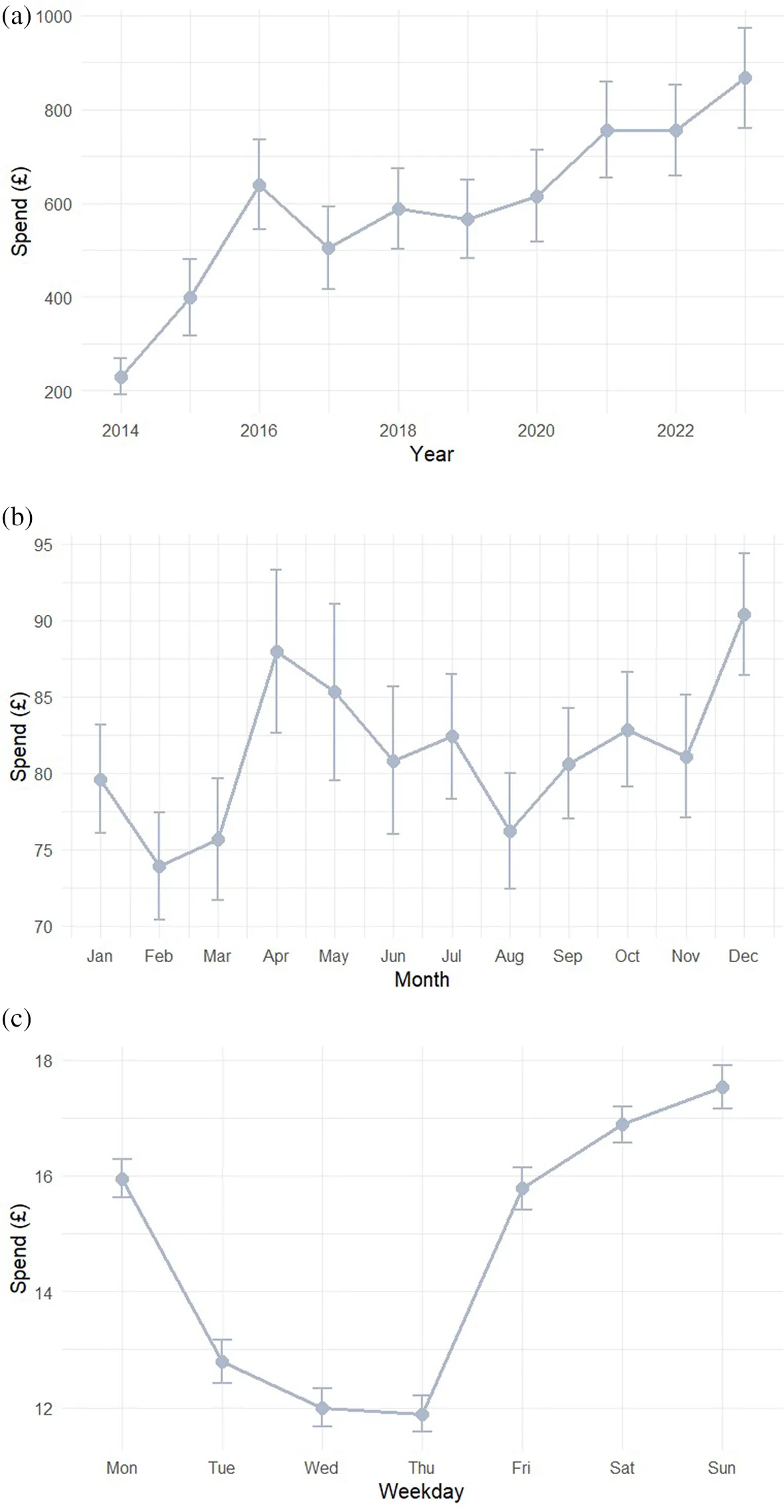
Average spend by participants (with standard deviation) by a) year, b) month, and c) weekday.

The dataset also includes discounts; a discount rate is applied to 15,466 items. The mean discount is 11.52% (SD = 2.53), the minimum discount is 0.15%, and the largest discount is 20%.

### Products and categories

Prior research has examined purchasing within specific product categories, including dairy, ^26^ seafood, ^27^ fruit and vegetables, ^6^ ^,^ ^28^ ^,^ ^29^ plant-based foods, ^30^ or alcoholic drinks. ^31^ To our knowledge, shopping data has not been used to examine purchasing patterns related to clothing, homeware, fuel, or other non-dietary/medication related items.

There are 55,176 unique products in this dataset, spread across a range of product groups. Each product is classified into one of 82 broad categories (e.g., produce, dairy) and 1002 subcategories (e.g., bananas, bread). Table 7 shows the top ten product categories based on total spend, and Table 8 shows the top ten categories based on total number of items purchased. In each case, the top three categories remain the same—produce, packaged grocery, and dairy—with other categories also remaining high, including ready meals, soft drinks, and health and beauty products. A summary of spend and number of items purchased for all categories is provided in Table 9. Anomalies (<10 items) have been removed, in addition to variables related to trading or business. Variable name refers to the category as it is labelled in the dataset (for ease of data requests). Quantities less than 5 are written as <5 to protect participant identities.

**Table 7.** Top ten categories by total spend.

Category	Spend (£)	Percentage (%)
Produce	133,954.41	10.78
Packaged Grocery	99,211.74	7.99
Dairy	97,894.07	7.88
Ready-to-eat Meals	76,155.46	6.13
Confectionery	64,420.83	5.19
Meat, Fish & Poultry	59,387.80	4.78
Soft Drinks	54,686.20	4.40
Health & Beauty	54,094.79	4.35
Cold Convenience	41,897.26	3.37
Frozen Foods	39,275.19	3.16

**Table 8.** Top ten categories by total items purchased.

Category	Total items	Percentage (%)
Produce	120,816	9.73
Packaged Grocery	75,250	6.06
Dairy	58,461	4.71
Confectionery	45,923	3.70
Ready-to-eat Meals	35,198	2.83
Soft Drinks	34,064	2.74
Crisps & Snacks	24,117	1.94
Health & Beauty	21,332	1.72
Cold Convenience	19,941	1.61
Chilled Desserts	19,573	1.58

**Table 9.** Total spend and quantity of purchases made within in product category.

Theme	Category	Variable name	Spend (£)	Quantity
Fruit & Vegetables	Produce	Produce	133,954.41	120,816
Food Cupboard	Packaged Grocery	Packaged Grocery	99,211.74	75,250
	Ethnic	Ethnic	10,494.87	8146
	Tinned items	Canned Goods/Preserv	294.85	185
Dairy	Dairy	Dairy	97,894.07	58,461
	Ultra-High-Temperature Milk	UHT Milk	5281.31	3511
	Cheese Counter	Cheese Counter	457.51	200
Snacks	Confectionery	Confectionery	64.420.83	45,923
	Crisps & Snacks	Crisps & Snacks	32,623.21	24,117
Fresh Foods	Cold Convenience	Cold Convenience	41,897.26	19,941
	Chilled Desserts	Chilled Desserts	27,682.76	19,573
	Deli Counter	Deli Counter	468.04	301
	Fresh Foods	Fresh Foods	1.00	<5
Ready Meals & Meal Deals	Ready-to-eat Meals	Meal Solutions	76,155.46	35,198
	Food To Go	Food To Go	830.30	343
Drinks	Soft Drinks	Soft Drinks	54,686.20	34,064
	Hot Beverages	Hot Beverages	31,152.28	12,970
	Beers	Beers	31,569.70	7949
	Wines	Wines	36,553.71	5409
	Spirits & Fortified Wine	Spirits/Fortfd Wines	23,041.40	1620
	Kosher Wine	Kosher Wine	66.75	19
Health & Beauty	Health & Beauty	Health & Beauty	54,094.79	21,332
	Pharmacy	Pharmacy Senior Mgr	1180.18	360
	Cosmetics	Cosmetics	1403.61	227
Bakery	Bread, Cake & Morning Goods	Brd Cake & Mng Gds	21,622.35	18,924
	In-Store-Bakery: Bread and Cake	ISB Brd/Cke Rcpe	17,323.22	16,474
	Cake	Cake	7230.15	3591
	In-Store-Bakery: Bread and Cake	ISB Brd/Cke Sold	2570.20	1455
	In-Store-Bakery Ingredients	ISB Ingredients	224.55	208
Freezer	Frozen Foods	Frozen Foods	39,275.19	18,910
	Frozen Meat, Fish & Poultry	Frozen MFP	3907.89	1332
Meat	Meat, Fish & Poultry	Meat Fish & Poultry	59,387.80	16,143
	Bacon, Fish & Sausages	Bacon & Fsh/Ssges	15,430.65	6797
	Fish	Fish	14,129.12	4310
	Fish Counter	Fish Counter	494.71	110
	Meat/Fish Counter	Mest/Fish Counter	102.35	37
Home	Household	Household	33633.06	14016
	Homeshop	Homeshop	450.12	225
	Cards	Cards	7491.51	3565
	Home	Home	24,049.30	3242
	Electrical	Electrical	6781.73	486
	Entertainment	Entertainment	10,112.31	1084
	Leisure	Leisure	1121.33	238
	Accessories & Furniture	Accessries&Furniture	24.85	15
Children	Kids Shop	Kids Shop	15,249.04	6252
	Toys	Toys	6842.22	705
Pets	Petcare	Petcare	12,028.10	4376
Clothes	Fabric	Fabric	11,111.28	2531
	Childrenswear	Childrenswear	15,103.85	1982
	Womenswear	Womenswear	22,548.01	1842
	Menswear	Menswear	5724.66	471
	Beeline Jewellery	Beeline Jewellery	427.50	70
Stationary & News	Stationery	Stationery	3550.78	1517
	News & Mags	News & Mags	3150.75	1491
	Postage Stamps	Postage Stamps	140.50	56
Tobacco	Tobacco	Tobacco Snr Manager	5522.39	723
Vehicles	Car Wash	Car Wash	324.96	56
	Petrol	Senior Managr.Petrol	11,998.04	9064
Seasonal	Seasonal	Seasonal	9165.10	2909
Other/Unknown	Trading Systems	Trading Systems	0.00	2841
	Smart boxes and Carrier Bags	Smrtbxs & Crrier Bgs	1230.92	6504
	Flowers	Floral	8747.15	1657
	Sainsburys To You	Sainsburys To You	3700.12	1453
	Sales Exclude	Sales Exclude	4281.41	1452
	JSR Sellable	JSR Sellable	2510.72	1127
	Hot Curry/Chicken	Hot Curry/Chicken	1565.65	861
	Food Prep Sellables	Food Prep Sellables	2419.55	654
	Forecourt Lines	Forecourt Lines	561.66	199
	Deliveries Cloth OL	Deliveries Cloth OL	106.75	177
	ETU/Non JS Cards	ETU/Non JS Cards	2468.74	108
	In Store Advertising	In Store Advertising	72.50	61
	Icon Live	Icon Live	146.75	47
	JSR Standard	JSR Standard	84.45	36
	JSR Ordered	JSR Ordered	73.50	25
	Cards & Wrapping	Cards & Wrap Conv	29.70	14
	CustomerOrdering NSE	CustomerOrdering NSE	132.16	10
	Electronic Gift Card	Electronic Gift Card	360.50	8
	ICW Watches	ICW Watches	86	7
	Groc/Froz/Confect	Groc/Froz/Confect	0.43	<5
	Retail Pharmacy	Retail Pharmacy	11.25	<5
	Mobiles & Accessories	Mobiles & Acc	1.98	<5

These purchase patterns reflect the central role of produce in household spending (e.g.,), ^28^ supporting the use of loyalty card data to study fruit and vegetable intake. The frequent purchase of ready meals, soft drinks, and confectionary highlights opportunities to examine processed food consumption (e.g.,). ^32^ Additionally, “health and beauty” may provide insights into health-related behaviours, including the purchase of over-the-counter medications and personal care products, ^8^ including women’s health related purchases. ^9^ Category-level purchasing data also provide valuable opportunities to examine dietary transitions, ^30^ to assess overall dietary diversity, ^3^ or to develop dietary phenotypes by clustering individual purchasing behaviours into distinct customer profiles. ^33^

We also carried out (case insensitive) string searches on product names to examine the presence of additional categories of interest in the dataset; these are shown in Table 10. These offer insight into nuanced behaviours that may not be captured by standard food group classifications, which can be restrictive, ^1^ and could be used to explore health-conscious purchasing, ethical consumption, brand preferences, and financial constraints, for example.

**Table 10.** Additional shopping categories of potential interest, with explanation.

Search term	Number of items	Explanation
Organic	6938	May reflect health, environmental, or ethical food choices.
Low Fat	650	Indicates health-conscious purchasing, particularly related to weight management or heart health.
“Taste the Difference” ^1^	26,047	Represents premium brand choices, useful for studying spending patterns and food quality preferences.
Basics	6713	Suggests budget conscious shopping, relevant to socioeconomic or food insecurity research.
Gluten Free	252	Indicates dietary restrictions or health-motivated choices (e.g., coeliacs disease, gluten sensitivity).
Free Range	5113	May reflect ethical consumption and concern for animal welfare.
Vegan	1441	Captures plant-based dietary preferences or ethical/environmental motivations.
Sugar Free	382	May indicate health-conscious behaviour, particularly related to diabetes or calorie control.
Fairtrade	10,131	May reflect ethical consumerism and awareness of global trade practices.

^1^ “Taste the Difference” is Sainsbury’s premium own-brand product range, offering higher-quality food and drink items compared to its standard and value ranges.

### Store location

The dataset includes geographical identifiers for store location, allowing for analyses on a spatial level. Each transaction is linked to a store code, which is mapped to different levels of Office of National Statistics (ONS) geographies: Output Area (OA), Lower Layer Super Output Area (LSOA), and Middle Layer Super Output Area (MSOA) (ONS, 2025). An OA is the most granular level (~40 households are covered per OA), followed by LSOA (~650 households) and MSOA (~3000 households). ^34^ These geographies are based on those used for the 2021 Census Data, and may be valuable for additional linkage to neighbourhood-level measures (such as the Index of Multiple Deprivation). The transactions in this dataset were made across 726 OAs, 703 LSOAs, or 637 MSOAs.

To generate these geographical identifiers, ALSPAC requested a separate list of store addresses and postcodes from Sainsburys. These were used to assign ONS geographies via postcode lookup. Further recoding may be required within the TRE to identify specific store locations for analysis. At this stage, we are unable to comment on the geographic distribution of stores represented in the dataset, as this requires additional linkages (i.e., of store postcodes) that were out of the scope of this work. However, the spatial coverage of these data could support research into area-level deprivation and inequalities, ^35^ proximity and access to food retail, ^36^ or geographic variation in spending. ^6^

Figure 9 shows the number of distinct Output Areas visited by participants. On average, participants shopped within 12.9 distinct areas (SD = 11.8); the maximum areas visited was 68, and the minimum was 1. On average, participants visited a single area 21.7 times (SD = 80.5); with the maximum number of visits to a single area being 1669, and the minimum being 1.

**Figure 9.**
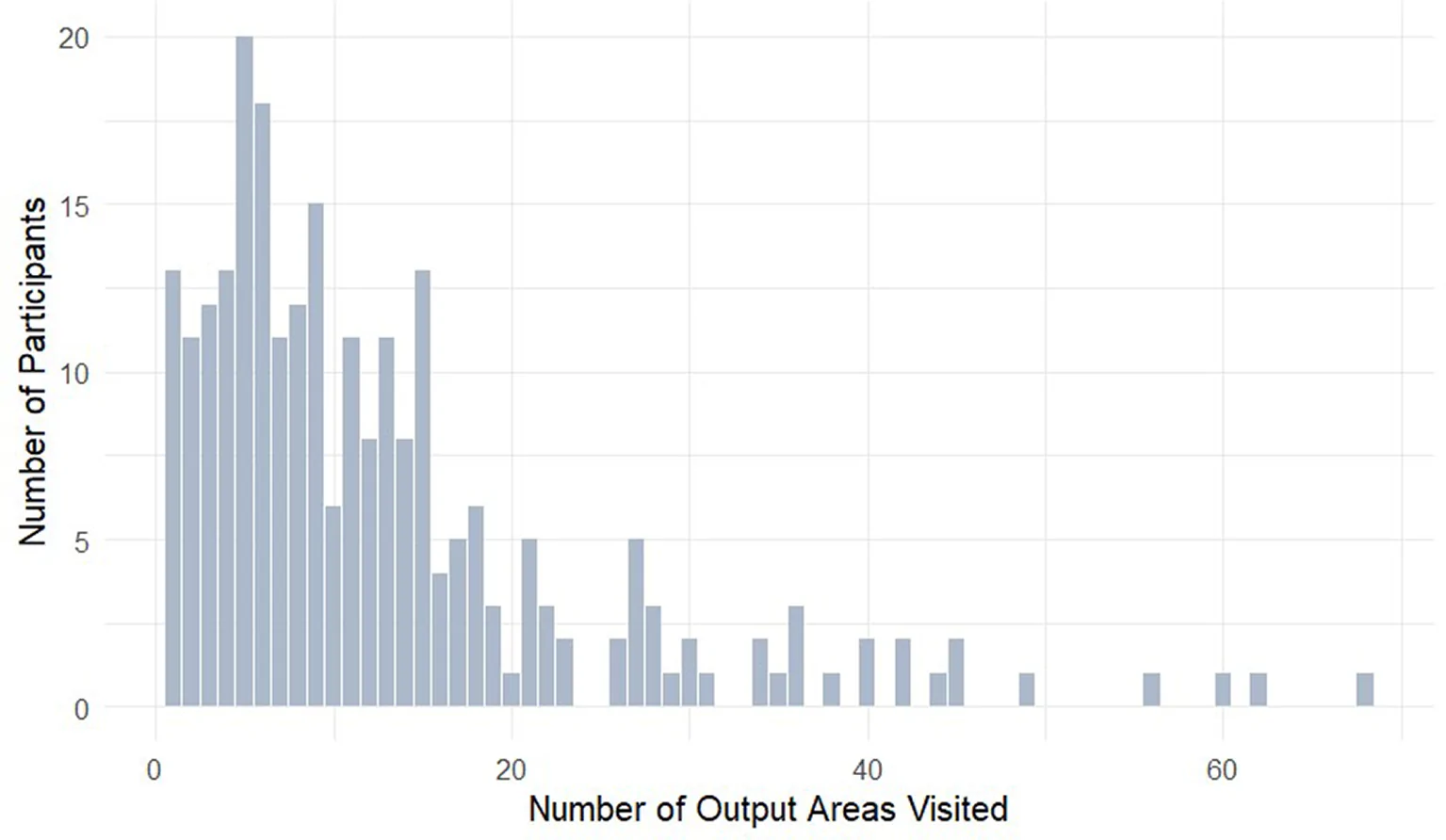
Number of Output Areas (OA’s) visited by participants.

Figure 10 shows the number of distinct Lower Layer Super Output Areas (LSOA) visited by participants. On average, participants shopped within 12.8 distinct areas (SD = 11.5); the maximum areas visited was 67, and the minimum was 1. On average, participants visited a single LSOA 21.9 times (SD = 81.9); with the maximum number of visits to a being 1669, and the minimum being 1.

**Figure 10.**
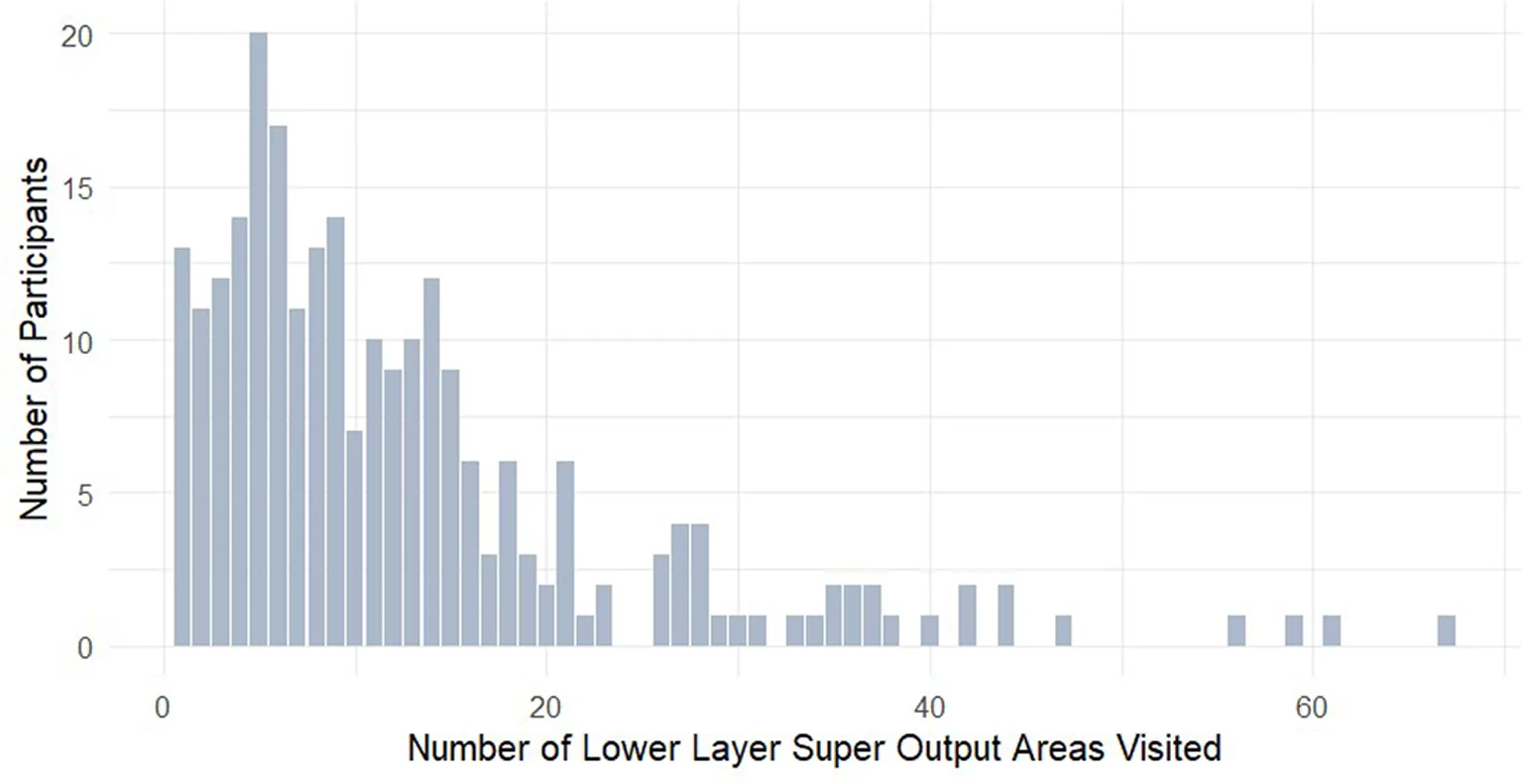
Number of Lower Layer Super Output Areas visited by participants.

Figure 11 shows the number of distinct Middle Layer Super Output Areas (MSOA) visited by participants. On average, participants shopped within 12.2 distinct areas (SD = 10.9); the maximum areas visited was 60, and the minimum was 1. On average, participants visited a single MSOA 22.9 times (SD = 83.8); with the maximum number of visits to a single MSOA being 1669, and the minimum being 1.

**Figure 11.**
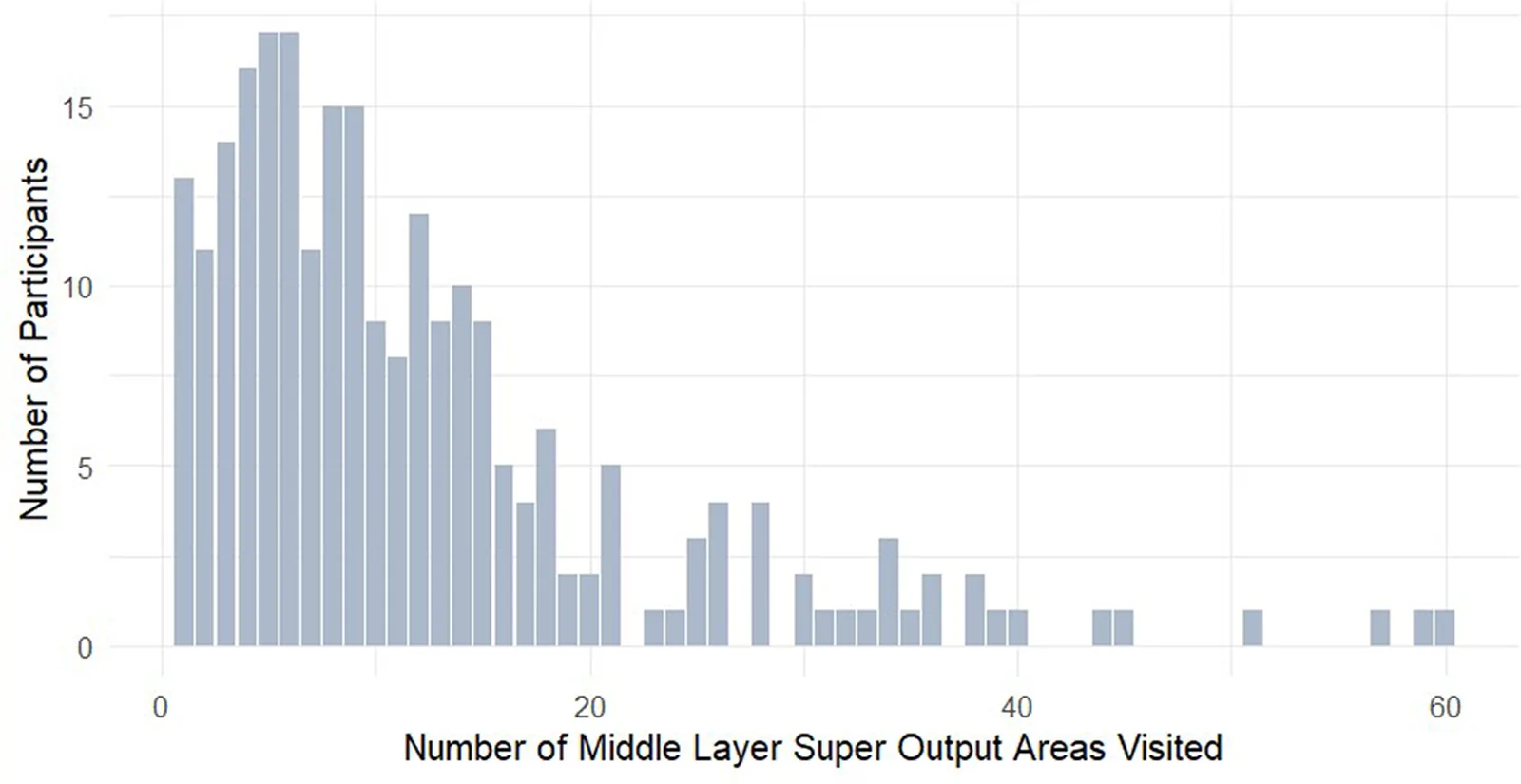
Number of Middle Layer Super Output Areas visited by participants.

## Discussion

This data note describes the results of a linkage of participant’s Sainsburys Nectar Card loyalty card records into the ALSPAC database. In total, 457 participants reported owning a loyalty card and provided details for at least one of five schemes—Sainsbury’s Nectar, Tesco Clubcard, Morrisons More, Boots Advantage, and Co-op Membership—including 258 consenting to linkage of Sainsbury’s records. Of these, data was successfully linked for 244 G1 participants—a linkage rate of 94.6%. This novel dataset offers several strengths for research, providing objective purchase data based on real-world records. The data is extremely detailed, capturing precise information on product types, spend, and frequency of purchases over a ten-year period. In addition, the longitudinal and retrospective nature of the records will allow researchers to examine historic purchasing behaviours and link them to high quality ground-truth data collected over the life span, to evaluate purchasing, dietary habits, nutritional intake, and subsequent health outcomes.

The linked cohort is largely representative of recently active participants in the wider G1 cohort, although there are some differences in financial comfort and educational attainment. Females are over-represented, in line with general response patterns for ALSPAC in this age group. More generally and linked to the ALSPAC eligibility criteria, participants are all approximately the same age, and the majority are of white ethnicity. Other features—being more likely to be highly educated and of higher socioeconomic status—are well-documented biases in ALSPAC participation. ^17^ Importantly, when compared with other recent ALSPAC response patterns, the 244 participants who consented to loyalty card linkage do not appear markedly different in their demographic or socioeconomic profile. This suggests that willingness to provide loyalty card data is broadly consistent with existing engagement behaviours within the cohort, rather than reflecting a distinct subgroup. Nonetheless, as in other loyalty card studies, potential biases should be acknowledged, including the tendency for participants to be female, ^32^ older, ^37^ white, ^37^ or have differences in reporting specific health factors (e.g., “normal” BMI, higher diet quality). ^38^ Together, these factors must be considered when generalising findings to the UK population and beyond.

While shopping data offers many insights on its own, ^12^ the real value of this dataset comes from the ability to link it with more than 30 years of historic ALSPAC data. This includes health measures, biomarkers, lifestyle questionnaires, and socioeconomic indicators, as well as linked health records, administrative data (such as education, employment, and social care records), and environmental measures. This allows us to examine a very wide variety of questions, exploring how what people buy relates to diet quality, health outcomes, financial security, wellbeing, and other changes throughout the life course.

Overall, our linked dataset makes it possible to address questions that would be very challenging using typical participant-reported data collection methodologies as these would likely be impacted by recall and/or social desirability biases and be very burdensome to collect beyond very short time windows. The examples in Figure 12 illustrate the types of analyses these data can support.

**Figure 12.**
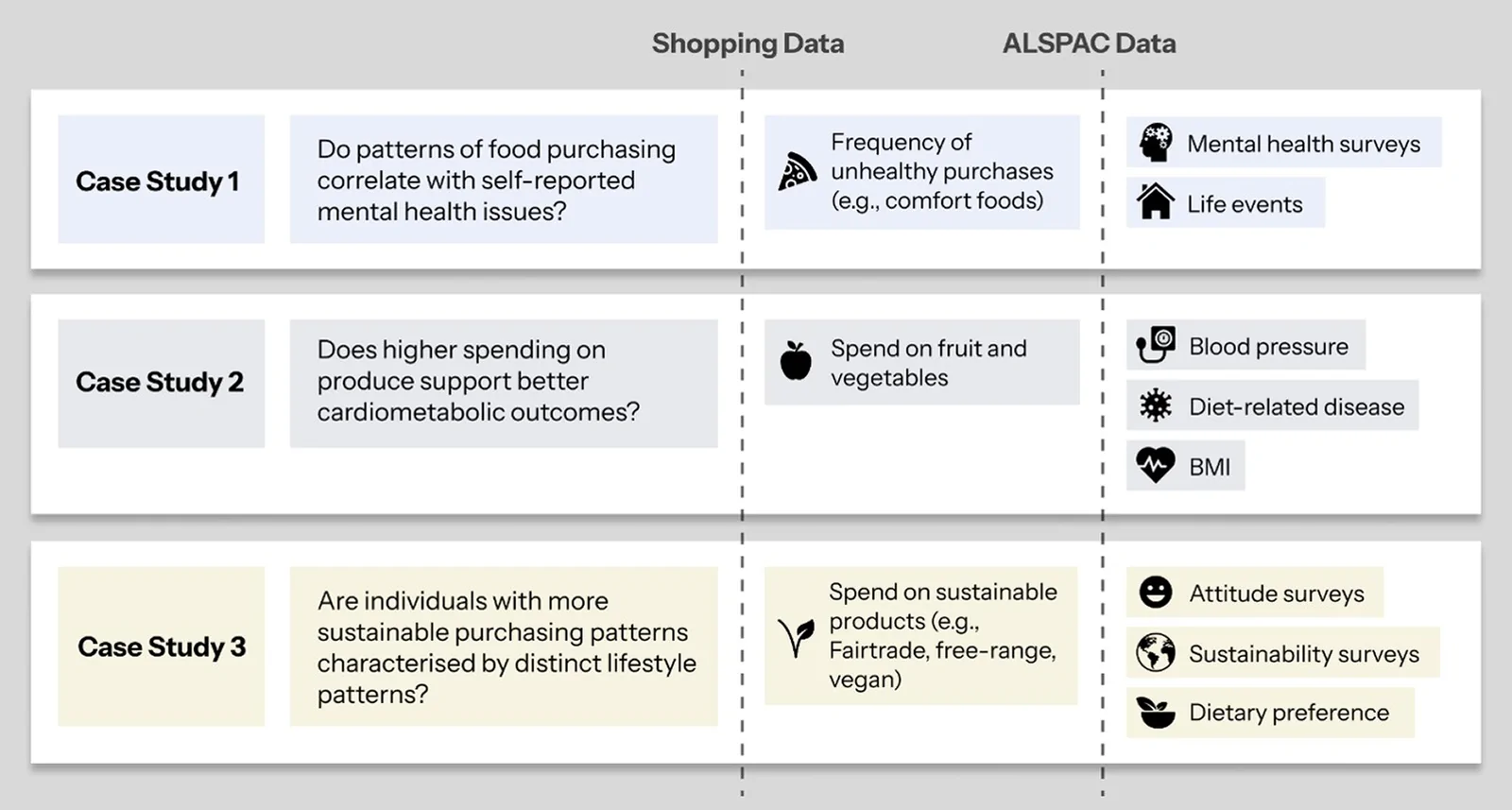
Example case studies made possible using the linked dataset.

We recommend that researchers use the ALSPAC study website to identify measures of interest for specific research questions ( http://www.bristol.ac.uk/alspac/researchers/our-data/).

### Limitations

However, there are also limitations to this dataset. As with many forms of observational digital footprint data compared to other more deliberate data collections (e.g., surveys), the purchase records are not consistent across participants. Some individuals have only a small number of recorded purchases, whereas others have thousands spanning many years. Similarly, the observation window varies substantially between participants. In addition, not all participants purchase items from every product category (e.g., some may rarely or never purchase food), meaning that analyses focused on specific product categories will necessarily rely on subsamples with sufficient purchasing frequency and duration. This selective inclusion may introduce additional bias.

The dataset also lacks product attributes beyond basic purchase information. While nutritional information is a key example (e.g., calories, fats, proteins), other attributes such as ingredients, allergens, sustainability indicators, or processing level are also unavailable. Consequently, future research would benefit from linkage to external product composition databases to derive the attributes of interest, in order to enrich the dataset further. This is common practice in shopping data research. ^5^ ^,^ ^39^

The data also reflects various household compositions (e.g., families versus single adults), and we do not know whether items were consumed by only the study participant or other members of the household. There may also be multiple cardholders within the household. Future work will be carried out to validate the records as a measure of food intake, leveraging additional surveys within ALSPAC (e.g., food-frequency questionnaires). ^40^ Relatedly, the data only captures purchased items, telling us nothing about actual consumption, waste, or food consumed from other retailers and eateries. To account for this, we plan to extend linkage to the remaining four retailers that participants consented to sharing their data for.

Lastly, some limitations relate specifically to the linkage process rather than shopping data specifically. Although linkage was conducted securely and in line with ethical requirements, the process was resource-intensive for the linkage team and required management of a large number of individual files, risking the introduction of processing error.

## Conclusion

This study demonstrates the successful and secure linkage of Sainsbury’s loyalty card data into the Avon Longitudinal Study of Parents and Children for 244 participants, creating a novel dataset of detailed, longitudinal purchasing behaviour. These records provide objective insights into diet and behaviour over a ten-year period and, when combined with ALSPAC’s rich databank, offer unique opportunities to explore relationships with health.

While the current dataset has limitations—including slight over-representation of females, high educational attainment, missing purchases from other retailers, and the absence of direct nutritional and descriptive attributes—it represents a pioneering example of integrating supermarket purchase data into a longitudinal cohort. This work establishes a foundation for future research, including potential expansion to additional retailers and participants, to further enhance understanding of dietary behaviours and public health outcomes.

## Data access

Access to this dataset can be granted to researchers upon reasonable request by submitting a proposal to the ALSPAC website ( http://www.bristol.ac.uk/alspac). It is recommended that researchers first familiarise themselves with the ALSPAC access policy and browse existing projects on the research proposal database, before submitting a proposal for consideration by the ALSPAC executive committee. Interested parties may email alspac-data@bristol.ac.uk with additional queries.

Data are provided within the study’s TRE to researchers as Stata or Excel files.

## Code availability

The code used to generate the variables in this data note is available on GitHub ( http://www.github.com/Digital-Footprints-Lab).

## Ethics statement

The collection and linkage of shopping data was approved by the ALSPAC Ethics and Law Committee on the 26 ^th^ July 2022 (Project ID: B3200). Written informed consent for the use of all data collected was obtained from participants following the recommendations of the ALSPAC Ethics and Law Committee at the time. Participants can contact the study team at any time to retrospectively withdraw consent for their data to be used. Study participation is voluntary and during all data collection sweeps, information was provided on the intended use of data.

## Publication consent

We confirm that we have consent to publish and share the data in this study.

## Data Availability

These data are available to approved researchers upon reasonable request, following submission of a proposal via the ALSPAC website ( http://www.bristol.ac.uk/alspac). Data are stored and provided within the study’s Trusted Research Environment.
