# Supplementary material for: Data Note: Linking Shopping Transaction Data into the Avon Longitudinal Study of Parents and Children
Source: Wellcome Open Res. 2026 Oct 8;11:821. [Version 1] doi: 10.12688/wellcomeopenres.27761.1 (PMC13760144; doi:10.12688/wellcomeopenres.27761.1)
Supplement: Peer review history

# Reviewer report: Reviewer response for version 1

**DOI:** 10.21956/wellcomeopenres.30552.r177985

**Published:** 2026-11-12

**Authors:** Roberto Mansilla

**Affiliations:** 1 University of Nottingham, Nottingham, UK

This Data Note outlines the collection and integration of Sainsbury's Nectar loyalty card data from ALSPAC participants. The authors obtained the data through Subject Access Requests for 244 G1 participants, covering the period from July 2014 to February 2024. The authors compare this group to other recent G1 participants, discuss observed shopping patterns, and provide information on how researchers can access the data.

 It’s a really valuable resource, and the work with participants before the linkage is excellent. That said, a few things need fixing. I couldn’t reproduce some of the key summary figures. Some conclusions about representativeness don’t match the tables. And some details about how the data were produced are missing.

***Major comments***

- Total spend is given as £1,531,686 (page 11), but the Table 7 percentages seem to be based on a total of about £1.24m (e.g., Produce £133,954 = 10.78%). The categories in Table 9 also add up to about £1.24m. Please explain the difference.
- In Table 8, Produce is 120,816 out of 658,375 items, which I make 18.4% rather than 9.73%. Please say which denominator was used.
- The wider G1 denominator for sex (33,419) is larger than the whole G1 cohort (14,701 in Figure 1). Please check which group this refers to.
- The abstract says the group is “predominantly made up of individuals with higher educational attainment”. If this means A levels or above (88%), it would help to say so, because only 45% have a degree. 
- The paper describes the differences in financial comfort as “small” (e.g., page 5). But 31% vs 46% are “living comfortably”, which is quite a big gap, and the linked group is actually less well off than the wider G1 group. Please revisit this wording, ideally with a formal test.
- Does Figure 7 use the full period from July 2014 to February 2024? If so, some months appear more often than others: July to January ten times each, but March to June only nine times, and February 2024 only partly. Because the number of active cards grows over time, could this affect the monthly totals? If so, it might be worth showing the monthly patterns using only complete years (2015–2023) and adjusting for the number of active cards. Also, the peak on Mondays is a bit unexpected for UK grocery shopping; adding a short explanation will help.
- The dataset has a store ID and the store’s area (Table 4), but no information on store type. Some categories in Table 9, such as “Sainsburys To You”, “Deliveries Cloth OL”, “Petrol” and “Forecourt Lines”, suggest that online orders and petrol station purchases may be included. Could the authors clarify which channels and store types are covered, and how they record online orders? A variable for store type or channel would be very useful for users, especially for any analysis based on store location (online vs in-store purchase behaviours).

***Minor comments***

1. Table 1: The wider cohort GCSE figure (636/3,576) looks like 17.8% rather than 28%, and the column adds up to 110%.
2. Tables 5 and 6: Both add up to 68,342 transactions rather than 68,392, possibly because of gaps between groups (e.g., a £5.50 transaction doesn’t fit “£1–£5” or “£6–£10”). Please use groups with no gaps or explain the rationale for the current groups.
3. Other small mismatches:
  - 263 vs 258 “consenting” (Results vs Discussion)
  - 24 variables in the text but 20 in Table 4
4. The consent is described as “written” in one place and electronic in another. Please make this consistent.
5. References 4 and 39 are the same paper.

Are sufficient details of methods and materials provided to allow replication by others?

Partly

Is the rationale for creating the dataset(s) clearly described?

Yes

Are the datasets clearly presented in a useable and accessible format?

Partly

Are the protocols appropriate and is the work technically sound?

Partly

Reviewer Expertise:

Social data science and consumer behaviour analytics, using large-scale transaction and loyalty card data to study food purchasing, diet and health.

I confirm that I have read this submission and believe that I have an appropriate level of expertise to confirm that it is of an acceptable scientific standard, however I have significant reservations, as outlined above.

# Reviewer report: Reviewer response for version 1

**DOI:** 10.21956/wellcomeopenres.30552.r177991

**Published:** 2026-11-12

**Authors:** Paul Longley

**Affiliations:** 1 University College London, London, England, UK

It is very welcome to see the development of new procedures to link conventional survey instruments to new sources of consumer data, and Sainsbury’s willingness to participate in this work is very much to be applauded. A generation ago, creating the equivalent corpus of consumer panel data would have entailed a major diary-based research endeavour which, apart from the huge expense, would have been vulnerable to the classes or recall error, telescoping of purchasing into incorrect timeframes and sample attrition (e.g. https://journals.sagepub.com/doi/10.1068/a161221). This said, an obvious caveat is that the records do not provide a panopticon view of all purchasing behaviour across all major chains and independents, requiring acknowledgment that the linked data are fundamentally incomplete (see https://www.sciencedirect.com/science/article/pii/S0969698921002162).

 There may be some issues that require some attention. The authors suggest that Monday is the most popular grocery shopping day, which does not coincide with my own observations. Reported spend also seem curious, with figures highest on Friday through Sunday (which I would expect) followed by Monday (which I would not). The authors also report that they have been unable to georeference the establishments at which shopping takes place. Whilst this is a pity in itself, it made me wonder whether the authors have included online purchasing from ‘dark’ stores that have georeferences (my store loyalty card records if I buy things from Greenford, but no shoppers ever go there) and whether some purchases (but not all – perhaps just those made online) made on non-banking are first recorded on the next banking day (a Monday).

 The absence of geocoding also means that store format is not captured, limiting the potential to analyse shopping trip purpose (main shop, top up) and issues of sensitivity to prices – something that the treatment of discounts also obfuscates.

 The 82 product categories are detailed but not chosen by the researchers, which limits some analysis (e.g. of drinks with controversial histories of health claims, such as SunnyD)  but I think it reasonable that enlightened retailers may consider highly granular product categories commercially sensitive.

 The authors consider sources of bias but are less clear about how it may operate. We might not be overly concerned about the apparent over-representation of females, since they are known to be more likely than males to register for store loyalty programmes (with Boots Advantage card being perhaps the most skewed example), regardless of who collects the points or consumes the groceries. But I missed any comment on whether multiple card ownership within a single household was accommodated – so how many records are of individuals and how many are households?

 I think these are important caveats to analysis, but part of a rich agenda into the methods and data sources that are becoming prevalent in social investigation. This work is an important contribution that has delivered a valuable linked data source, and the authors are very much to be commended for their successful engagement with a major data provider and for delivering an important data product.

Are sufficient details of methods and materials provided to allow replication by others?

Yes

Is the rationale for creating the dataset(s) clearly described?

Yes

Are the datasets clearly presented in a useable and accessible format?

Yes

Are the protocols appropriate and is the work technically sound?

Partly

Reviewer Expertise:

Geographic Information Science

I confirm that I have read this submission and believe that I have an appropriate level of expertise to confirm that it is of an acceptable scientific standard.

# Reviewer report: Reviewer response for version 1

**DOI:** 10.21956/wellcomeopenres.30552.r173811

**Published:** 2026-10-29

**Authors:** Emma Wilkins

**Affiliations:** 1 University of Leeds, Leeds, England, UK

This paper describes a novel and valuable linkage between loyalty card data and ALSPAC. This new linked data allow for several limitations of existing consumer purchase data research to be addressed via linkage of purchase data with detailed demographic, lifestyle and health data over time. I enjoyed reading this paper, and think the linked data is very valuable.

 I have three major comments, followed by some very minor points requiring clarification.

**Major Comments**

*Incomplete or Unclear Methods Information*

 The Technical Pilot Section:- This section describes a technical pilot study performed for a subset of participants. A number of details are unclear: The details of *how* the subject access request was made are unclear. What is the ‘participant documentation’? Who made the subject access request? Did participants sign the form, share this with ALSPAC, and then ALSPAC sent to Sainsbury’s? Is the electronic signature the participant’s signature? “Files were encrypted” – what files? Sent to whom and by whom?

 No information is provided in the methods section for the ‘main’ linkage (i.e. what was done following the pilot). Some of this methods information appears to be included in the results section, but should be brought up to the methods section.

 No information is provided on any quality screening or processing after receipt of the data. However, the results allude to several processing steps. For example, Table 9 suggests that some recoding/renaming of categories took place. And results also state that ‘anomalies were removed’ – please give details on how these were identified, with examples. Furthermore, did you screen and/or impute product weight information? From my experience product weight data tends to be quite incomplete and the quality is poor. Product weight data can also be obtained for produce from several data fields, since the actual weight purchased is often available in the transaction data, alongside an indicative weight per product (which is typically the average weight per item and not the actual weight purchased). Please clarify how product weight was handled after receipt from the retailer.

*Repeated reference to consumption/nutritional behaviours*

 The manuscript makes repeated reference to the fact that purchase data can be used to understand consumption patterns or nutritional behaviours (or words to that effect). However, loyalty cards typically capture purchases for a household rather than for a single person, and it is not possible to make inferences about what was actually consumed by the G1 linked person. Please revise the wording throughout, particularly in the abstract, which reads as though the linked purchase data are for individuals not (potential) households.

 Relatedly, the manuscript states “People can encompass not only individual card holders, but also households (i.e., if card holders shop for other people).” – I think this sentence could be stated more clearly and elaborated on to make the limitations more evident.  

 The fact that we can't infer consumption from these data should also be added as a limitation in the conclusion.

*More data needed to understand the limitations of not capturing all purchases from all retailers*

 The authors allude to the fact that the data doesn’t capture all purchases made by participants (which will include purchases from other grocery retailers, convenience stores and out of home), but this could be made clearer and stated explicitly as a limitation in the discussion. 

 Additionally, in the results section, it would be helpful to see some data to help understand how ‘complete’ the data may be in terms of capturing all purchases.   For example, the authors report descriptive data for the number of transactions per shopper over the loyalty card useage window. However, since the loyalty card usage window varies per shopper, it would be helpful to normalise this to a time period. For example authors could report descriptive statistics for the number of transactions per year or per month. This would give a better sense of how complete the data are likely to be in terms of capturing all purchases.

 In the limitations section, it may be useful to reference recent work exploring how purchase thresholds can be applied to the data to improve agreement between purchase nutrients and consumed nutrients: https://www.nature.com/articles/s41366-026-02107-1

**Minor comments**

*Abstract*

- GDPR - give full name on first mention
- Clarify who made the subject access requests and to whom
- 82 categories – would be helpful to give one or two examples in addition to 'produce' to understand the scope of the data. 
- Consists of participants of similar age – give data, how old?
- Conclusion – first sentence needs reviewing, as this isn’t a full sentence.
- It’s not clear to me from the abstract that the data are from loyalty cards, which typically capture shopping at the household level, rather than individual level. This needs to be clear, and some mention of this limitation needs to be stated in the abstract.
- As above, please consider removing language that suggests we can measure nutritional intake.
- “novel opportunity for future research into dietary habits, nutritional intake, and subsequent health outcomes.” Data are broader than just food and drink, yet the abstract focuses on nutrition. This sentence also needs to be expanded.

*Backgroud*

 “So far, most studies” – not clear which studies you refer to. Is this specifically studies that have used loyalty card data?

*Results*

- 658,375 items in total – is this individual items sold? I assume it is, based on the large number. But this could be interpreted as the number of unique products (identifiable by sku or GTIN codes).
- “On average, participants used their cards for 2054 days (SD = 1256.13), or around 5.6 years”. – on first reading, I interpreted this to mean that transactions occurred on 2054 individual days. However, based on the scale of the number, I think you mean that transactions linked with a given card spanned a period of 2054 days (wherein transactions might occur e.g. 1-2x per week on average within that period). Please clarify.
- Table 4 *- *Unit price – is this price paid by consumer? Does this include any discounts or is this pre-discounted proce?
- Table 9 – Fresh foods category - < 5 items seems implausible. Is this a coding error? Or is this a little-used category? Might be worth adding a comment about this.

*Other*

- “Sainsburys” should be “Sainsbury’s” throughout.
- Heading - “The avon longitudinal study of parents and children” – this should be capitalised.
- Stray space before a closing bracket. 
- Nectar Card – sometimes ‘Card’ is not capitalised. Please be consistent.
- "The frequent purchase of ready meals, soft drinks, and confectionary highlights opportunities to examine processed food consumption (e.g.,)" – should an example be given here?
- Reference 4 and 39 applear to be duplicates.

Are sufficient details of methods and materials provided to allow replication by others?

Partly

Is the rationale for creating the dataset(s) clearly described?

Yes

Are the datasets clearly presented in a useable and accessible format?

Partly

Are the protocols appropriate and is the work technically sound?

Yes

Reviewer Expertise:

Use of non-traditional data (e.g. consumer purchase data) to understand the environment and how it shapes healthy behaviours.

I confirm that I have read this submission and believe that I have an appropriate level of expertise to confirm that it is of an acceptable scientific standard, however I have significant reservations, as outlined above.
